# Optimization of Camera and Radar Placement for Sensor Fusion and Ball Tracking in Sports

**DOI:** 10.3390/s26092809

**Published:** 2026-04-30

**Authors:** Dylan Kamstra, Johan Pieter de Villiers

**Affiliations:** Department of Electrical, Electronic and Computer Engineering, University of Pretoria, Pretoria 0002, South Africa; pieter.devilliers@up.ac.za

**Keywords:** covariance intersection, triangulation, sensor placement, optimization

## Abstract

The placement of sensors in an environment can significantly impact the sensing performance of a sensor fusion system. In this paper, the placement of cameras and radars is optimized based on the log determinant of the fused measurement noise of the sensor measurements. This is achieved by mapping the measurements into 3D Cartesian space and applying covariance intersection to obtain a final measurement distribution, which is taken as the measurement noise. The method was tested against random initial placements and optimization runs of sensors for a system that is intended for ball tracking in sports. The particular use case involves the tracking of a cricket ball for the purpose of match evaluation and assisted umpiring. However, in principle, the method is applicable to any sensor placement problem in which the objective is localization and tracking. The results indicate an improved root mean squared error for the optimized sensor placements, which in turn implies a reduction in the measurement noise covariance.

## 1. Introduction

Optimal sensor placement is a vital aspect of system design in any system where sensor measurements are used for any kind of inference, such as state estimation or classification. This is especially true in sensor fusion systems such as cameras, radars, and lidars, as the placement of these types of sensors affects the characteristics of the measurements, in the form of the sensors’ resolution, potential occlusions, coverage of the desired area, and redundancy. In addition, some sensor types may complement the weaknesses of other sensor types. In many circumstances, the placement of sensors has been left to expert human judgment, yet this is subject to the expert’s experience, which can be misguided.

Common applications of optimal sensor placement are attempts to increase the field of view (FOV) of a given area constrained by the number and type of cameras [1,2,3]. This type of application has a well-defined objective; however, the problem is NP-hard as there are many placements of the cameras that can yield the same result. An analogous problem to maximizing the FOV is tracking indoor movement [4]. These applications look at a simplified map of the region of interest (ROI), where while the sensors are modelled in 3D, the optimization is done in a 2D bird’s-eye view (BEV) of the ROI, which ties into their objective function.

In many applications, objects of interest are triangulated from their observations in images, most notably in tracking-based tasks, to obtain valuable 3D state information about the object. This can be used for reconstruction purposes, such as in motion capture [5]. Here, cameras are allowed to vary within a constrained range, limited by the nature of the problem. This can be extended to the optimal view of human pose [6]. Furthermore, the problem of triangulation involves the problem of overlapping coverage of camera sensors [7], which is important to consider in a static environment and influences the number of cameras used.

A different approach to sensor placement is to work with the covariance of the sensor measurements. The aim is to decrease the volume of the ellipsoid of the measurement covariance matrix. In [8], the problem is reformulated as a sensor selection problem, where the measurement function is linear in the state space. The log determinant of the covariance matrix is used as the minimization criteria to select the correct sensors. Alternatively, the trace of the steady-state error covariance of a Kalman filter (KF) can be used to place the sensors in nodes in a graph [9,10].

To optimize camera placement, the most commonly used algorithms do not have access to the closed-form expression of the objective function derivative with respect to sensor pose. As such, algorithms such as Simulated Annealing (SA) [5,11], Particle Swarm Optimization (PSO) [12], and genetic algorithms (GA) [7] are used, which explore the surface of the loss functions. Greedy-based algorithms are another approach [13], where sensors are placed based on a voting scheme. Although these algorithms produce good results, they suffer from large computational loads and long execution times. An alternative approach is to use Gaussian processes (GP) [14] to find sensor configurations that maximize the mutual information between the chosen sensor locations and the locations not chosen. Not all the sensors need to be chosen at the start, as in [15], where sensors are added one by one to ensure that the minimum number of sensors is used.

In [16], error propagation is presented for computer vision problems, including curve fitting, local feature extraction, and exterior and relative orientation. However, it does not address the problem of triangulation. In [17], an algorithm is given to find pairs of points to obtain an initial triangulation, which can then be used to improve triangulations from multiple sensors. Many attempts have been made to find a fast, robust three-view camera triangulation algorithm [18,19,20], yet these are limited in the number of cameras that they can use, a number which is baked into the problem to be solved.

In this paper, a Gradient Descent (GD) algorithm is presented for minimizing a matrix norm of the measurement covariance (obtained by covariance intersection) for a system of cameras and radars by varying the position and orientation of the sensors. The measurement noise dominates the accuracy of tracking filters; minimizing the measurement noise will increase the performance of a filter that uses the sensors. This is achieved by fusing the measurements of the sensors in Cartesian space based on their inherent noise parameters. This is done through the use of covariance intersection (CI) [21]. CI has been used to fuse cameras and radars/lidars in simultaneous localization and mapping (SLAM) [22,23,24], as the cross-correlation between the sensor types is unknown.

The remainder of this paper is structured as follows. Section 2 provides a mathematical objective to the problem and presents the assumptions made. In Section 3, the sensor models and the fusion of their measurements are presented, while in Section 4 the basic GD algorithm is formulated and the constraints of the system are presented. In Section 5, the system is tested for different initial placements of the sensors, and the results are discussed. Lastly, Section 7 presents the conclusions and potential future work.

## 2. Problem Formulation and Assumptions

Given a space $R^{3}$ or set of coordinates in the space that must be observed by at least two sensors, either a camera and/or a radar, the problem can be formulated as finding the optimal sensor centre in the world coordinates c and orientation transformation matrix R, which transforms a state-space vector from world coordinates to sensor coordinates, where c is a point-like 3×1 vector representation of the sensor’s position (the focal point of the camera or the antenna location of the radar), while R is a 3×3 rotation matrix. Each sensor’s c and R are initialized from an initial set of offsets and orientations, $\bar{c}$ and $\bar{R}$, such that a matrix norm (the determinant, trace, or root mean square error (RMSE)) of the fused measurement covariance is minimized. The problem can be formulated as

(1) $$C_{opt},R_{opt}=\mathrm{argmin}\frac{1}{N}\sum_{x_{i}\in X}\mathrm{norm}\left[A\left(Y\left(C,R,x_{i}\right),C,R\right)\right]$$

where $X=\{x_{1},x_{2},\ldots ,x_{N}\}$, $x_{n}$ is a 3×1 position vector of a target/object, or a point of interest (POI), that is observed, for example, at sample time *n*. The measurement of $x_{n}$ is given by Y, which produces a set of measurement vectors $\left\{y_{n}^{\left(1\right)},y_{n}^{\left(2\right)},\ldots ,y_{n}^{\left(I\right)})\right\}$, where *I* is the number of sensors, and $y_{n}^{\left(i\right)}$ is a M×1 vector of measurements of $x_{n}$ made by the $i^{th}$ sensors. The set of offset vectors for the sensors is given by $C=\{c^{\left(1\right)},c^{\left(2\right)},\ldots ,c^{\left(I\right)}\}$, $R=\{R^{\left(1\right)},R^{\left(2\right)},\ldots ,R^{\left(I\right)}\}$ is the set of sensor rotation matrices, and *A* is the measurement fusion function that produces a 3×3 covariance matrix.

The algorithm for the measurement covariance intersection is discussed in Section 3.5, which shows how it is related to the rotation and position of each of the sensors; however, it is not difficult to see that the position and orientation will have an effect on the outcome of the fusion. It is important to note that $c_{0}$ and $R_{0}$ has the potential to play a vital role in the final values of C and R, depending on the number of sensors used, as this is an NP problem.

This formulation allows for an arbitrary number of sensors greater than two to be used, as a minimum of two sensors is required for the intersection of the measurements’ covariances to have a well-defined covariance matrix. However, this approach makes a basic assumption: that all the projections of the POIs in X onto the sensors will result in a successful fusion. This assumption is intuitive if the environment in which the fusion will occur is in an ROI, i.e., it is known that all the fusion will occur within a bounded area. Since two sensors cannot be in the same place, there will always be an information exchange between the sensors.

## 3. Covariance Fusion Theory

### 3.1. Camera Mapping

A camera can be modelled as having both intrinsic and extrinsic parameters. The extrinsic parameters are its position in the world coordinates c and rotation R. The intrinsic parameters are given by

(2) $$K=\left[\begin{array}{ccc} f_{x} & s & u_{0}\\ 0 & f_{y} & v_{0}\\ 0 & 0 & 1 \end{array}\right],$$

where $f_{x}$ and $f_{y}$ are the camera focal points for the *x*-axis and *y*-axis, *s* is the skew parameter, and $u_{0}$ and $v_{0}$ are pixel offsets so that the origin of the image is at the top-left corner. For the remainder of the paper, it is assumed that K is kept constant. This is set as the focal length and the translation of the camera are intertwined, i.e., moving the camera backwards while increasing the focal length will negate each other; thus, keeping it constant removes it from the dynamic variables. This fact will come into use when constraining the offset of the cameras.

The complete mapping of a POI $x={\left[\begin{array}{ccc} x & y & z \end{array}\right]}^{T}$ onto the image is given by

(3) $$\lambda \left[\begin{array}{c} u\\ 1 \end{array}\right]=K\left[R|-Rc\right]\left[\begin{array}{c} x\\ 1 \end{array}\right]=K\left(R\left(x-c\right)\right),$$

where the pixels are defined up to a scale factor λ, $u^{T}={\left[\begin{array}{cc} u & v \end{array}\right]}^{T}$ are the position of the pixel, and homogeneous coordinates are used for the 3D POI and pixels. It is the division of λ in (3) that moves the POI u from the 3D domain to the 2D-pixel domain. The use of homogeneous coordinates in x is such that the mapping can be combined into a matrix P. Expanding (3) out and incorporating the division by λ, one obtains

(4) $$u={\left[\frac{P_{\bar{1}}{\left[x^{T},1\right]}^{T}}{P_{\bar{3}}{\left[x^{T},1\right]}^{T}}\;,\frac{P_{\bar{2}}{\left[x^{T},1\right]}^{T}}{P_{\bar{3}}{\left[x^{T},1\right]}^{T}}\right]}^{T},$$

where $P_{\bar{i}}$ is the *i*th row of P, given by

(5) P=K[R|−Rc].

Equation (4) shows how important R and c are in the mapping process.

### 3.2. Radar Mapping

Similar to the camera, the radar is defined in space by a rotation and a translation. The mapping of a POI x from world coordinates to radar coordinates r=(r,θ), where *r* is the range and θ is the azimuth, is a non-linear mapping of

(6) $$\begin{array}{cc} r & =||R(x-c)||, \end{array}$$

(7) $$\begin{array}{cc} \theta & =\arctan\frac{R_{\bar{2}}\left(x-c\right)}{R_{\bar{1}}\left(x-c\right)}, \end{array}$$

where $R_{\bar{1}}$ and $R_{\bar{2}}$ are the first and second rows of the rotation matrix R respectively. This mapping goes from 3D Cartesian coordinates to 2D polar coordinates, where the elevation of the POI is lost.

For completeness, the elevation of a POI is given by

(8) $$\rho =\arccos\frac{R_{\bar{3}}\left(x-c\right)}{r}.$$

### 3.3. Camera Covariance

The mechanism by which noise enters the camera measurement process in (3) is the result of several influences. Even if the calibration of the camera is performed perfectly, the quantization of the light intensities owing to pixelization and noise in the electronics of the camera at the moment an image is taken will perturb the pixel values. We defer to the central limit theorem, and make a simplifying assumption where the addition of several measurement imperfections results in an additive Gaussian noise model; therefore, the perturbed pixel value $\tilde{u}$ is given by

(9) $$\tilde{u}=u+w,$$

where w is zero-mean independent Gaussian noise with covariance matrix $\Sigma_{u}$. Since cameras lose depth information, when projecting the pixel point back into 3D space, the coordinate x exists along the ray

(10) $$x=\lambda R^{T}K^{-1}\left[\begin{array}{c} \tilde{u}\\ 1 \end{array}\right]+c.$$

If the value of λ is known, then the 3D POI can be estimated. Equation (10) represents an affine transformation of the pixel point, which can be applied to a Gaussian distribution; however, care must be taken in defining the values in Σ.

Consider the Gaussian distribution

$$g=N({\left[\begin{array}{c} x\\ y\\ z \end{array}\right]}_{g},\Omega_{g}^{-1}),$$

where $\Omega_{g}$ is a 3×3 precision matrix given by

$$\Omega_{g}=\left[\begin{array}{cc} \Sigma_{g}^{-1} & 0\\ 0^{T} & 0 \end{array}\right],$$

and $\Sigma_{g}$ is a 2×2 covariance matrix, and 0 is a 2×1 zero vector. The Gaussian distribution described by g can be seen as a 2D Gaussian distribution projected into 3D space, where the variance along the *z*-axis approaches infinity. This is why it is easier to work with the precision matrix, as the covariance matrix is undefined—this can be seen by noting that the determinant of the precision matrix is zero, and so its inverse matrix does not exist.

Now consider a similarly defined Gaussian distribution

$$h=N({\left[\begin{array}{c} x\\ y\\ z \end{array}\right]}_{h},\Omega_{h}^{-1}),$$

where $\Omega_{h}$ is defined as

$$\Omega_{h}=\left[\begin{array}{cc} 0 & 0^{T}\\ 0 & \Sigma_{h}^{-1} \end{array}\right].$$

This Gaussian distribution similarly has zero precision along the *x*-axis. Looking at the two Gaussian distributions g and h, it is clear that together they constrain the covariance matrix along all axes of 3D space, g constrains the variance in the xy plane and h constrains the variance in the yz plane. Taking the product of these two functions yields a Gaussian distribution of

$$N(\mu ,\Sigma^{\prime }),$$

where μ and Σ are given by

$$\begin{array}{cc} \Sigma^{\prime } & ={\left(\Omega_{g}+\Omega_{h}\right)}^{-1}\\ \; & ={\left[\begin{array}{ccc} \Omega_{g}^{\left[11\right]} & \Omega_{g}^{\left[12\right]} & 0\\ \Omega_{g}^{\left[21\right]} & \Omega_{g}^{\left[22\right]}+\Omega_{h}^{\left[11\right]} & \Omega_{h}^{\left[12\right]}\\ 0 & \Omega_{h}^{\left[21\right]} & \Omega_{h}^{\left[22\right]} \end{array}\right]}^{-1},\\ \mu & =\Sigma^{\prime }\left(\Omega_{g}{\left[\begin{array}{c} x\\ y\\ - \end{array}\right]}_{g}+\Omega_{h}{\left[\begin{array}{c} -\\ y\\ z \end{array}\right]}_{h}\right), \end{array}$$

where $\Omega_{\cdot }^{\left[ij\right]}$ represents the element in the *i*th row and *j*th column of $\Omega_{\cdot }$, and (−) is used to show that the value of this variable can be set arbitrarily as the corresponding values in the precision matrix are zero. Looking at $\Sigma^{\prime }$, it is clear that this matrix is well-defined, and that μ contains no redundant information. This can be scaled to an arbitrary number of low-dimensional Gaussian distributions projected up into the same higher-dimensional space.

Within the context of camera sensors, the ray coming from the camera center through the image point (given by applying the inverse intrinsic matrix to the pixel point) can be viewed in a similar light as projecting a 2D Gaussian distribution in the camera pixel space into the 3D world coordinates space, where the depth axis has zero precision.

In camera coordinates, the depth axis is given by the *z*-axis. The 2D Gaussian distribution is

(11) $$N(\left[\begin{array}{c} u\\ v \end{array}\right],\Omega_{u}^{-1}),$$

where $\Omega_{u}$ is the pixel precision matrix, the image coordinates in 3D are given by

(12) $$N(K^{-1}\left[\begin{array}{c} u\\ v\\ 1 \end{array}\right],\Omega_{x}^{-1}),$$

and

(13) $$\Omega_{x}=K_{2}^{T}\left[\begin{array}{cc} \Omega_{u} & 0\\ 0^{T} & 0 \end{array}\right]K_{2},$$

where the intrinsic parameter matrix results in addition owing to $u_{0}$ and $v_{0}$ in (2). This results in a translated covariance matrix in (13) which violates the fact that covariance matrices are only scaled and rotated. To resolve this, the upper left 2×2 matrix in K is used and the remaining elements are set to zero, denoted $K_{2}$.

Since the Gaussian is formulated in terms of precision, the matrices in (13) are multiplied using the identity

$${\left(A_{1}A_{2}\cdots A_{k-1}A_{k}\right)}^{-1}=A_{k}^{-1}A_{k-1}^{-1}\cdots A_{2}^{-1}A_{1}^{-1},$$

where $K^{-1}$ is bought into the matrix inversion in (13).

The formulation in (12) is, however, incomplete. In (12), the line that has an undetermined variance is the camera’s *z*-axis, not the ray defined in (10). To correctly align the Gaussian distribution, an additional rotation is applied to the precision matrix. Let the projected coordinate on the image plane in 3D space be defined as

(14) $$\overset{˚}{x}=\frac{K^{-1}{\left[\begin{array}{ccc} u & v & 1 \end{array}\right]}^{T}}{\left|\right|K^{-1}{\left[\begin{array}{ccc} u & v & 1 \end{array}\right]}^{T}\left|\right|}.$$

The rotation from $x^{\circ }$ to the *z*-axis basis function $e_{z}={\left[\begin{array}{ccc} 0 & 0 & 1 \end{array}\right]}^{T}$, i.e., $R_{\overset{˚}{x}}\overset{˚}{x}=e_{z}$, can be constructed by calculating the correct basis vectors. The new *z*-axis is given by

$$e_{z}^{\prime }=\frac{\overset{˚}{x}}{\left|\right|\overset{˚}{x}\left|\right|}.$$

The new basis vector for the *x*-axis is constructed by making $e_{x}$ perpendicular to the new *z*-axis basis function by

$$e_{x}^{\prime }=\frac{e_{x}-\left(e_{x}\cdot e_{z}^{\prime }\right)e_{z}^{\prime }}{\left|\right|e_{x}-\left(e_{x}\cdot e_{z}^{\prime }\right)e_{z}^{\prime }\left|\right|}.$$

Lastly, the new *y*-axis basis is found by taking the cross-product of the two new basis functions

$$e_{y}^{\prime }=e_{z}^{\prime }\times e_{x}^{\prime }.$$

The rotation matrix is then defined as

(15) $$R_{\overset{˚}{x}}=\left[\begin{array}{c} {\left(e_{x}^{\prime }\right)}^{T}\\ {\left(e_{y}^{\prime }\right)}^{T}\\ {\left(e_{z}^{\prime }\right)}^{T} \end{array}\right],$$

This formulation only allows for the Gaussian to be pitched and yawed, which will preserve the orientation of the covariance ellipse of the pixel noise, where the covariance ellipse or ellipsoid is defined as the *N* sigma constant covariance contour.

Applying this rotation matrix to (12), we get

(16) $$N(K^{-1}\left[\begin{array}{c} u\\ v\\ 1 \end{array}\right],{\left(R_{\overset{˚}{x}}^{T}K_{2}^{T}\left[\begin{array}{cc} \Omega_{u} & 0\\ 0^{T} & 0 \end{array}\right]K_{2}R_{\overset{˚}{x}}\right)}^{-1}),$$

which now has the major axis of the covariance ellipsoid aligned with the ray through the camera center and $\overset{˚}{x}$. When the rotation matrices enter into the matrix inversion, the rotation matrix property of its inverse, being its transpose, is used. Lastly, the rotation of the camera and the depth scale λ are applied to obtain the final precision matrix of

(17) $$\Omega_{c}=\frac{1}{\lambda^{2}}R^{T}R_{x_{n}}^{T}K_{2}^{T}\left[\begin{array}{cc} \Omega_{u} & 0\\ 0^{T} & 0 \end{array}\right]K_{2}R_{x_{n}}R,$$

which yields a Gaussian distribution of

(18) $$N(c,{\left(\Omega_{c}\right)}^{-1}),$$

where the mean of the Gaussian is replaced by the camera center. This is possible as the Gaussian distribution is now in world coordinates, and the mean can be any value along the ray in (10). Setting it to the camera center allows for the covariance matrix to describe any ray coming from the camera center if required.

It is possible to combine R and $R_{\overset{˚}{x}}$ into a single rotation by applying R in (14); however, care must be taken to ensure that the orientation of the camera axes will not change. This is only a concern if the pixel covariance is non-isotropic.

While the Gaussian in (18) will provide the correct covariance, λ is still not known. To find it, two of these covariance ellipsoids from different cameras can be intersected using an arbitrary λ, where the resulting Gaussian mean is a triangulated coordinate. From this coordinate, λ can be found by projecting the coordinate back onto each camera. The now correctly scaled covariance ellipsoids can then be intersected again to get a new mean and covariance. This can be performed iteratively to refine the results, similar to the iterative triangulation algorithms in [25].

**Figure 1:**
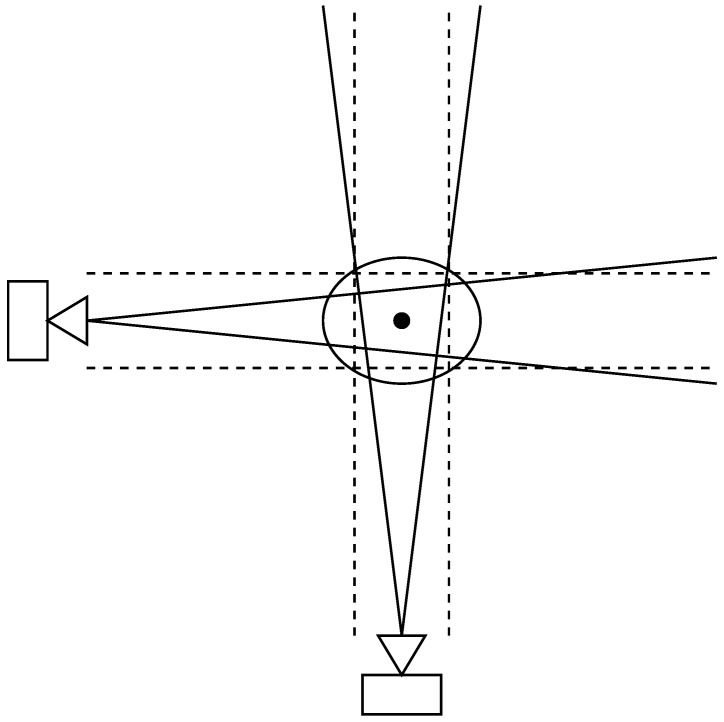
An exaggerated top-view of two cameras triangulating a POI, given by the circle. The solid lines show the one standard deviation ellipse, while the dashed lines are the approximation of the conic distributions. The resulting ellipse is shown.

While this process does provide a triangulated mean, the intersected covariance is an intersection of the individual camera covariance ellipsoids, which in turn approximate conic distributions. This can be seen in Figure 1. A particle approach is given in [26,27]; however, it does not yield a neat closed-form 3D probability distribution. The presented approach is able to easily scale to many cameras, as the process of finding the point of intersection of the different rays is handled through the use of the Gaussian distributions.

### 3.4. Radar Covariance

The conversion of a radar measurement to Cartesian space poses a greater challenge. The type of automotive radar used in this study only obtains two of the three spherical coordinates, namely range and azimuth. Similar to the cameras λ, the elevation of a POI can be found iteratively once a mean coordinate has been found by multiple sensor covariance intersection, using an initial guess of the elevation, such as the mean elevation of the POIs.

Since the range and azimuth measurements contain some noise, the unbiased converted measurements (UCM) approach in [28] is used to convert the radar measurements to 2D Cartesian coordinates. The assumed elevation ρ is then used to define a radar POI coordinate in 3D space,

(19) $$\overset{˚}{p}=\left[\begin{array}{c} x\\ y\\ z \end{array}\right]=\left[\begin{array}{c} e^{\sigma_{\theta }^{2}r/2}\cos\theta \sin\rho \\ e^{\sigma_{\theta }^{2}r/2}\sin\theta \sin\rho \\ r\cos\rho \end{array}\right],$$

where *r* and θ are the mean range and mean azimuth respectively, and $\sigma_{\theta }$ is the standard deviation of the azimuth. The inclusion of the scaling term is to remove the induced bias in the conversion between polar and Cartesian coordinates.

Similar to the camera’s initial covariance, the radar’s covariance has zero precision along the *z*-axis for an elevation of 0, that is, the covariance is constrained to the xy-plane. When the POI has a non-zero elevation, this plane needs to be tilted accordingly. This is accomplished by applying a rotation to the covariance matrix, which must ensure that the covariance matrix keeps the covariance between the *x*-axis and *y*-axis unchanged.

To achieve this, the axis is rotated to include the elevation, but also to set the azimuth of the measurement to 0. With an azimuth of 0, the *x*-axis must point towards the point $\overset{˚}{p}$

$$e_{x}^{\prime }=\frac{\overset{˚}{p}}{\left|\right|\overset{˚}{p}\left|\right|}.$$

The *y*-axis is chosen to remain on the xy-plane and is made perpendicular to $e_{x}^{\prime }$.

$$e_{y}^{\prime }=\frac{{\left[\begin{array}{ccc} -\sin\theta & \cos\theta & 0 \end{array}\right]}^{T}}{\left|\right|{\left[\begin{array}{ccc} -\sin\theta & \cos\theta & 0 \end{array}\right]}^{T}\left|\right|}.$$

Lastly, the *z*-axis is found using the cross product.

$$e_{z}^{\prime }=e_{x}^{\prime }\times e_{y}^{\prime },$$

and the full rotation matrix, which applies the elevation tilt, is given by

(20) $$R_{\overset{˚}{p}}=\left[\begin{array}{c} {\left(e_{x}^{\prime }\right)}^{T}\\ {\left(e_{y}^{\prime }\right)}^{T}\\ {\left(e_{z}^{\prime }\right)}^{T} \end{array}\right].$$

In this new coordinate frame, the UCM variance for a Gaussian noise distribution is given by

(21) $$\begin{array}{cc} R^{\left[11\right]} & =\frac{1}{2}\left(r^{2}+\sigma_{r}^{2}\right)\left[1+e^{-2\sigma_{\theta }^{2}}\right]\\ & +\left[e^{\sigma_{\theta }^{2}}-2\right]r^{2}\\ R^{\left[22\right]} & =\frac{1}{2}\left(r^{2}+\sigma_{r}^{2}\right)\left[1-e^{-2\sigma_{\theta }^{2}}\right]\\ R^{\left[12\right]} & =0, \end{array}$$

whereby setting the azimuth to 0, there is no cross-correlation between the *x*-axis and *y*-axis, since the azimuth information has been used in the construction of $R_{\overset{˚}{p}}$. The covariance of the point is initially given by

(22) $$\Sigma_{r}=\left[\begin{array}{cc} R^{\left[11\right]} & R^{\left[12\right]}\\ R^{\left[12\right]} & R^{\left[22\right]} \end{array}\right],$$

after which the tilt rotation is applied and the rotation R from world coordinates to radar coordinates is reversed

(23) $$\Omega_{r}=R^{T}R_{\overset{˚}{p}}^{T}\left[\begin{array}{cc} \Sigma_{r}^{-1} & 0\\ 0^{T} & 0 \end{array}\right]R_{\overset{˚}{p}}R,$$

which yields a final Gaussian distribution of

(24) $$N(R^{T}\overset{˚}{p}+c,{\left(\Omega_{r}\right)}^{-1}).$$

**Figure 2:**
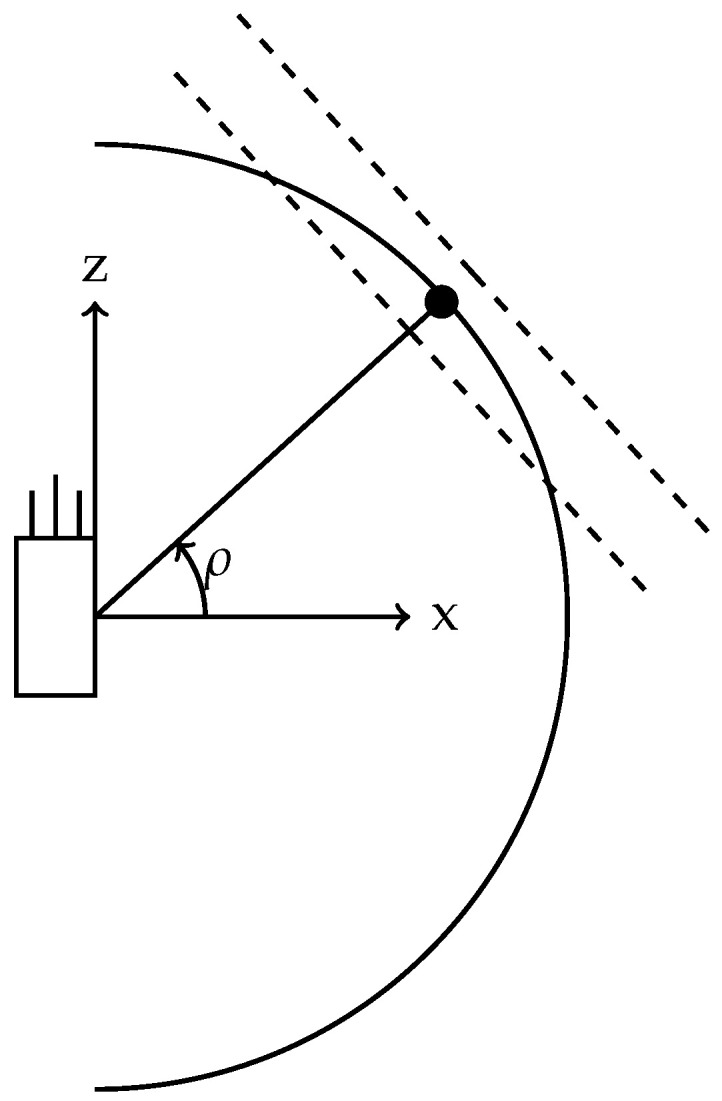
A side view of a radar and the tilted plane, at angle ρ, on which the rank-deficient covariance ellipse exists. The dashed lines show the one standard deviation of the ellipse at the POI for the radar sensor only.

Unlike the camera, combining R and $R_{\overset{˚}{p}}$ should not be performed to ensure that the orientation of $\Sigma_{r}$ is kept intact. Figure 2 shows how the plane is tilted and how the covariance ellipse is tangential to the range-elevation arc.

This conversion method is based on the elevation of the point being a scalar value. If a more refined estimate of the elevation is known, such as from a Kalman Filter (KF), then making use of the full 3D UCM or the 3D decorrelated unbiased converted measurement (DUCM) should be used, which can also easily be brought into the world coordinate system.

### 3.5. Covariance Fusion

When fusing the precision matrices from all sensors, the cross-correlation between sensors is unknown. Thus, covariance intersection (CI) is required. To avoid iterative weight selection (which complicates differentiation through the loss), we use uniform CI weights, i.e., each sensor weight is set to the reciprocal of the number of sensors, $w_{i}=1/S$.

Algorithm 1 provides the covariance-intersection fusion procedure. The algorithm takes as input the array of pixel values from each camera, the radars’ range and azimuth measurements, the assumed λ and ρ, as well as the extrinsic parameters of all sensors. For each sensor, the projected Gaussian distributions are computed using (18) and (24). The information-form CI with uniform weights is

(25) $$\Sigma \leftarrow {\left(\frac{1}{S}\sum_{\Omega_{i}\in \Omega_{s}}\Omega_{i}\right)}^{-1},$$

where *S* is the number of sensors and $\Omega_{s}$ is the set of sensor precision matrices. The fused mean is obtained by applying the same weight to the information vector:

(26) $$\mu \leftarrow \Sigma \left(\frac{1}{S}\sum_{\mu_{i},\Omega_{i}\in \mu_{s},\Omega_{s}}\Omega_{i}\mu_{i}\right),$$

where $\mu_{s}$ collects the individual sensor means. Equivalently, define the fused information matrix and vector as $\Omega =\frac{1}{S}\sum_{i}\Omega_{i}$ and $\eta =\frac{1}{S}\sum_{i}\Omega_{i}\mu_{i}$, then solve Ωμ=η and set $\Sigma =\Omega^{-1}$. With uniform weights, the fused mean equals the standard information-fusion mean, while Σ is inflated relative to naive fusion, as desired under CI. The use of uniform weighs is a simplification. If this algorithm is used merely for triangulation then the weights can be learnt through an iterative update approach. For the use in this application, having to update the weights introduces an optimization within an optimization; thus, it is left as uniform for the simplicity of the optimizer.

While the values of λ and ρ are not measured by the cameras and radars, respectively, they are required by Algorithm 1. As mentioned earlier, if this algorithm is used to perform covariance intersection, then λ and ρ can be iteratively estimated from an initial guess. Since Algorithm 1 is used as a step in sensor placement optimization, the true values of λ and ρ are used. This ensures the precision matrices have the correct scale and orientation so that the intersected covariance accurately reflects the noise in the triangulated coordinates.
**Algorithm 1** Covariance-intersection fusion**Input:** Pixels U, camera λ’s λ, radar measurements (with assumed elevations) M, Cartesian camera centers $C_{c}$, Cartesian radar centers $C_{r}$, camera rotations $R_{c}$, radar rotations $R_{r}$
**Output:** Fused mean μ, covariance matrix Σ
  1:$\mu_{s}\leftarrow$ empty array  2:$\Omega_{s}\leftarrow$ empty array  3:**for all** {u, λ, c, R} in {U, λ, $C_{c}$, $R_{c}$} **do**  4: Acquire $\overset{˚}{x}$ using (14)  5: Construct $R_{\overset{˚}{x}}$ using (15)  6: Compute $\Omega_{c}$ using (17)  7: Append c to $\mu_{s}$  8: Append $\Omega_{c}$ to $\Omega_{s}$  9:**end for**10:**for all** {$m_{r}$, c, R} in {M, $C_{r}$, $R_{r}$} **do**11: Acquire $\overset{˚}{p}$ using (19)12: Construct $R_{p^{\circ }}$ using (20)13: Compute $\Omega_{r}$ using (23)14: $\mu_{r}\leftarrow R^{T}\overset{˚}{p}+c$15: Append $\mu_{r}$ to $\mu_{s}$16: Append $\Omega_{r}$ to $\Omega_{s}$17:**end for**18:Compute Σ using (25)19:Compute μ using (26)20:**return** μ, Σ


It should be noted that the intersected covariance matrix can be rank-deficient when the zero-precision axes of multiple sensors align (e.g., cameras viewing along a common line through the POI, or radars whose zero-precision axes lie in a common plane).

## 4. Gradient Descent Approach and Constraints

### 4.1. Constraints and Considerations

While the sensor centers are needed in Cartesian coordinates to perform a linear mapping of a POI onto the sensor plane, applying GD to Cartesian centers will result in undesired behavior, owing to how uncertainty scales with distance. To illustrate this point, the Cartesian size of the covariance ellipsoid for the cameras is proportional to the λ, which itself is proportional to the distance that the point is from the camera center. Thus, points closer to the camera will have a lower covariance ellipsoid of the image plane than points further away. This makes intuitive sense. Optimizing the camera’s centers in Cartesian coordinates will result in the cameras moving closer to the POI coordinate mean in order to minimize measurement uncertainty. This is not practically achievable as cameras need to be a set distance from the ROI in order to keep the ROI within the FOV of the cameras.

Thus the sensor centers must be constrained. A useful approach will be to have the position of the sensors depend on some angle from the center of the ROI, which will be the main parameter on which GD is applied. This would imply moving the sensors along a predefined trajectory or parametric function such that the sensors’ FOVs remain centered on the center of the ROI. This implies that the sensors’ rotations need to be adjusted accordingly.

This makes the sensor rotation matrix a function of the sensor center. The rotation matrix can be constructed by pointing one of the axes, defined as the *a*-axis, towards the mean of the POIs, $\bar{x}$.

$$e_{a}^{\prime }=\frac{\bar{x}}{\left|\right|\bar{x}\left|\right|}.$$

**Figure 3:**
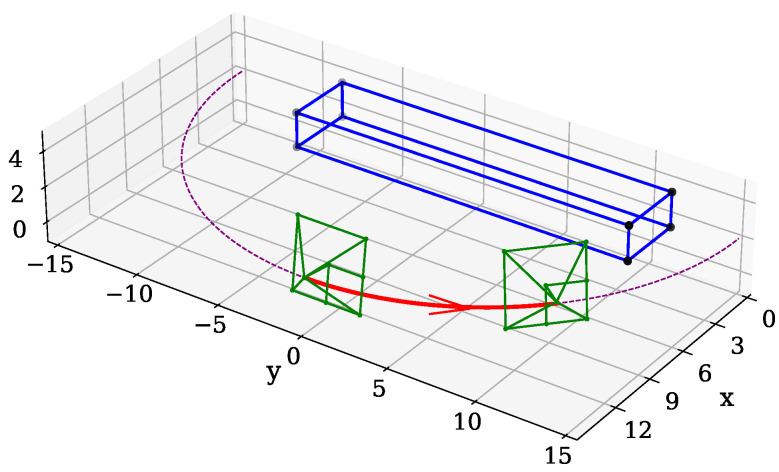
The blue rectangle represents the ROI, the purple dotted line is the path that the sensors are allowed to move on, the red arrow shows the movement of the sensor in one iteration step, and the green shows the centre and image plane of the sensor. Note that the orientation of the sensor changes in only pitch and yaw. The horizontal lines in the sensor’s plane, show two of the three axes of the sensor.

The second axis can be chosen to be parallel to the world coordinate xy-plane,

$$e_{b}^{\prime }=\frac{{\left[\begin{array}{ccc} -{\left(e_{a}^{\prime }\right)}_{y} & {\left(e_{a}^{\prime }\right)}_{x} & 0 \end{array}\right]}^{T}}{\left|\right|{\left[\begin{array}{ccc} -{\left(e_{a}^{\prime }\right)}_{y} & {\left(e_{a}^{\prime }\right)}_{x} & 0 \end{array}\right]}^{T}\left|\right|},$$

where ${\left(\cdot \right)}_{x}$ and ${\left(\cdot \right)}_{y}$ are the *x* and *y* values of (·). The final axis is then obtained using the cross-product

$$e_{c}^{\prime }=e_{a}^{\prime }\times e_{b}^{\prime },$$

and the rotation matrix is constructed,

(27) $$R_{s}^{\left(a\right)}=\left[\begin{array}{c} {\left(e_{a}^{\prime }\right)}^{T}\\ {\left(e_{b}^{\prime }\right)}^{T}\\ {\left(e_{c}^{\prime }\right)}^{T} \end{array}\right],$$

where the *a*-axis is aligned with the *z*-axis for the camera, and the *x*-axis for the radar, and the *b*-axis is aligned with the *x*-axis and the *y*-axis for the camera and radar respectively. This will point the sensors towards the mean of POIs in the ROI, using only a pitch and a yaw on the orientation of the sensors. An example is given in Figure 3, where a sensor’s center is set at a specific range and height, while being allowed to vary in azimuth.

One point of concern is the inversion of the sum of precision matrices to obtain Σ in Algorithm 1. If the condition number of this matrix is large, it will be harder to invert. This can be problematic, but it is unavoidable. Another point of consideration is that different noise factors between the sensors will result in different step sizes within the GD algorithm, as the level of contribution to the final covariance will depend on the noise parameters of the sensors. A method to circumvent this is discussed later.

### 4.2. Gradient Descent

The choice to use GD as the optimization algorithm is to showcase the ability of the CI-triangulation algorithm to have a useable derivative. Any optimization scheme can be used here to find the optimal placement of the sensors.

The loss function for the GD algorithm is given in Algorithm 2. It takes as arguments the POIs, and the camera and radar centers that can exist in any coordinate space. Since Algorithm 1 requires Cartesian sensor centers, the camera and radar centers are converted to xyz coordinates. These are then used to construct the rotation matrices for the sensors, which completes the extrinsic parameters of the sensors. The POIs are then projected onto each sensor using (3), (6) and (7). Furthermore, the λ and ρ for each of the POIs are also saved. In the initial pass, the mean values of λ and ρ for each camera and radar are used as an estimate, these can also be chosen based on the users knowledge of the environment. This allows Algorithm 1 to obtain a triangulated point, from which an updated estimate of λ and ρ are found for each POI. These estimates is then passed to Algorithm 1 for a second time to acquire a finer estimate of the covariance matrix, from which the loss for the POIs is calculated, using one of the metrics from Table 1, and averaged. During tests, the two-step algorithm with an initial mean λ and ρ was always able to match the triangulated point of the CI-triangulation where the true values of λ and ρ were used. Further testing showed that the λ can be arbitrarily set, so long as the initial values of λ were the same for all the cameras, as it only scales the camera covariance without changing the mean line. For ρ, an initial value that is within the expected range of elevation is required for a two-step approach; if not, multiple iterations are required for convergence to occur.
**Algorithm 2** Covariance fusion loss function with iterative λ and ρ estimation**Input:** 3D points X, camera centers $C_{c}$, Radar centers $C_{r}$, mean camera depth $\bar{\lambda }$, mean radar elevation $\bar{\rho }$
**Output:** Loss *l*
  1:$C_{c}^{\prime }\leftarrow$ conversion of $C_{c}$ to Cartesian coordinates  2:$C_{r}^{\prime }\leftarrow$ conversion of $C_{r}$ to Cartesian coordinates  3:$R_{c}\leftarrow$ alignment rotation matrices for cameras using (27), with $\bar{x}$ as the mean environment point  4:$R_{r}\leftarrow$ alignment rotation matrices for radars using (27), with $\bar{x}$ as the mean environment point  5:l← empty array  6:**for all x ∈ X do**  7: U← empty array {Camera projections}  8: Λ← empty array {Camera depths}  9: **for all** camera in cameras **do**10:  Project x to camera: obtain image point u (3)11:  Append u to U12: **end for**13: M← empty array {Radar projections}14: $P_{\rho }\leftarrow$ empty array {Radar elevations}15: **for all** radar in radars **do**16:  Project x to radar: obtain ${\left[r,\theta \right]}^{T}$ using (6)–(8)17:  Append ${\left[r,\theta ,\bar{\rho }\right]}^{T}$ to M18: **end for**19: $(\hat{\mu },\hat{\Sigma })\leftarrow$ covariance fusion of U, array of $\bar{\lambda }$, M, $C_{c}^{\prime }$, $C_{r}^{\prime }$, $R_{c}$, $R_{r}$ with these values20: For each camera, re-project $\hat{\mu }$ to get estimated $\hat{\lambda }$21: For each radar, re-project $\hat{\mu }$ to get estimated $\hat{\rho }$22: Replace $\bar{\rho }$ with $\hat{\rho }$ in M23: $(\hat{\mu },\hat{\Sigma })\leftarrow$ covariance fusion of U, $\hat{\lambda }$, M, $C_{c}^{\prime }$, $C_{r}^{\prime }$, $R_{c}$, $R_{r}$ with estimated λ and ρ24: $l_{x}\leftarrow \mathrm{lossFunction}\left(\hat{\Sigma }\right)$25: Append $l_{x}$ to l26:**end for**27:l←mean(l)28:**return** *l*


The loss function in Algorithm 2 can vary, with different functions minimizing different criteria. Table 1 provides a list of some potential loss functions. These are functions that convert a matrix to a scalar value, namely, the determinant, trace, and RMSE of a 3×3 matrix. Geometrically, the determinant of a covariance matrix is a representation of the volume of the covariance ellipsoid, while the trace is the total variation of the distribution, disregarding the correlation between variables. The RMSE is a measure of the error of the triangulated coordinate.

Because of the nature of the loss function and the fusion algorithm, it is recommended to use an instantiation of one of the tools in the class of *auto-diff* algorithms to obtain an estimate of the derivative of the loss function.

## 5. Results

The simulation of the sensor placement optimization is challenging owing to the large number of initial conditions, which include the sensor positions, the sensor noise values, and the number and type of sensors used. This is further exacerbated by a large set of POIs within the ROI for which the sensors must be optimized.

**Figure 4:**
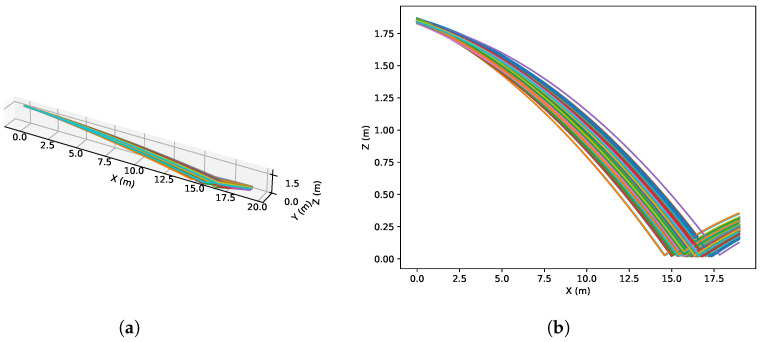
Paths of the simulated balls, where each colored line is a simulated ball. (**a**) shows the 3D paths of the balls, while (**b**) shows the side view of the balls.

The algorithm is tested on a cricket ball tracking use case, which is typically used for assisted umpiring and match analysis in sports broadcasting. The ball is modelled using equations of motion, including drag and lift forces, as well as spin. The spin is modelled using the equations from [29,30]. When the ball touches the ground, the velocity of the *z*-axis is inverted and multiplied by 0.5, which is meant to represent the energy lost during the bounce of the ball, called the coefficient of restitution. Figure 4 shows 200 simulated trajectories, which will be used as the set of POIs (135 POIs per delivery), where Δt was set to 1/180 for a max simulation time of 0.75 s. The initial positions and velocities of the balls were set using Gaussian distributions,

$$x_{0}=N\left(\left[\begin{array}{c} 0\\ 0\\ 2.0 \end{array}\right],\left[\begin{array}{ccc} 0.5 & 0 & 0\\ 0 & 0.1 & 0\\ 0 & 0 & 0.01 \end{array}\right]\right),$$

$$v_{0}=N\left(\left[\begin{array}{c} 0\\ 27\\ -3.8 \end{array}\right],\left[\begin{array}{ccc} 1 & 0 & 0\\ 0 & 1 & 0\\ 0 & 0 & 0.1 \end{array}\right]\right),$$

to introduce randomness into the paths of the balls. The positions of the balls are offset so that their mean is at the origin of the world coordinates. This makes positioning and constraining the sensors easier.

The sensors are positioned using cylindrical coordinates, where only the azimuth of the sensors is changed using GD. The range and height of the cameras are set to 25 and 10 m, respectively, while the radar range and height are set to 15 and 0 m.

As noted earlier, the range of the camera can change and still yield the same image, so long as the focal length of the camera also changes proportionally. To ensure that the ball will always be in the camera image, the intrinsic parameters of the camera are set to

$$K=\left[\begin{array}{ccc} 800 & 0 & 960\\ 0 & 3600 & 540\\ 0 & 0 & 1 \end{array}\right].$$

For the radar, the max range is set to 30 m with a max azimuth angle of $60^{\circ }$. This, too, is set so that the ball will always be viewed by the radar.

For the environment, it is assumed that there are no occlusions by the players; occlusions are uncommon in cricket broadcasting. Furthermore, it is assumed that the cameras are placed on the roof of the stadium and that the radars can be placed on the ground.

It was found that if a single optimizer was used on different types of sensors, those with a large variance in a certain dimension failed to be optimized, and the optimizer focused its efforts on the accurate sensor types in that dimension. Consequently, each sensor position is individually optimized according to the covariance matrix of its sensor type. This does present complications since the surface of the loss function will change per iteration step, as one group of sensors acts independently from the other and changes the surface of the loss function in an unexpected way. This complication can, however, be mitigated by increasing the number of optimization iteration steps and by updating only one sensor type per iteration step. The use of different optimizers ensures that each sensor type is properly placed while lowering the effects that the sensor noises have on the movement of the sensors.

The chosen optimizer is the ADAM optimizer [31], which will adapt the learning rates for each sensor type, mitigating the complication of different sensors having different noise values. ADAM is initialized with $\beta_{1}=0.9$, $\beta_{2}=0.999$, $\epsilon =1\times 10^{-8}$, and with a learning rate of α=0.02 for all sensor types. The use of ADAM also removes the need to change the optimizer when changing the minimization criteria, as different minimization criteria will require different learning rates to provide optimal results if stochastic GD (SGD) is used.

The noise of the camera is chosen to be a zero-mean Gaussian distribution with a standard deviation of three pixels. This is meant to encompass the noise in the projection process and the noise in detecting the ball within the image. The radar range and azimuth noise are set as a zero-mean Gaussian distribution with a standard deviation of 0.01 meters and 0.15 rad respectively, which is indicative of a mm-wave radar. These are noise values are indicative of what has been experienced using a mm-wave radar and cameras in cricket ball tracking.

**Figure 5:**
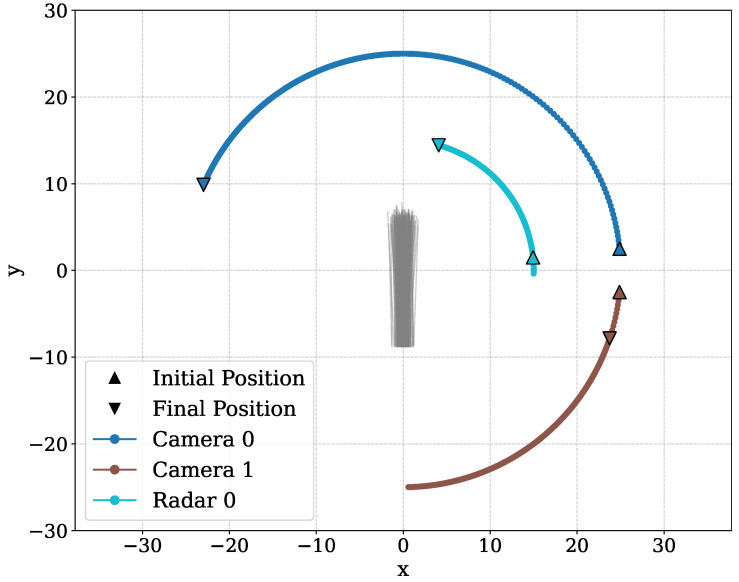
A BEV of the movement of the sensors for a one radar, two camera system, where the grey is the simulated balls. The determinant is the loss function utilized in this setup. Each dot represents a GD iteration step. Interestingly, the camera moves back onto itself, which is caused by the changing surface of the loss function at each second iteration.

To test the optimization scheme, a Monte Carlo simulation is performed where the initial orientation of the sensors is changed for each trial. A total number of 4500 trials is performed, and the number of iteration steps is set to 500, which, in testing the system, is long enough for the different minimization criteria to stabilize. To initialize the sensor types, each sensor is given a random angle θ∈[0,2π) such that no two sensors in the same sensor type share the same angle. This is performed for different sensor configurations, where the number of cameras and radars is varied. The results are shown in Table 2, where, if the sensors are positioned such that the CI-triangulation is unable to triangulate a point, the run is excluded. This is predominately for radar-only setups, due to the *z*-axis having a large variance value, and the triangulation struggling due to an estimate of ρ being used initially. For radar–camera, or purely camera, setups, above 90% of the runs are used, but for purely radar setups, the percentage drops to 80% for three and four radars, and to 40% for two-radar setups. This shows the utility of cameras in such a system, as one camera and one radar are able to easily triangulate a point. An example of the sensor movement is shown in Figure 5 for when the determinant is the metric in the loss function.

## 6. Discussion

The final RMSE values show that, as expected, as the number of sensors used increases, the RMSE decreases. Furthermore, within each subgroup, i.e., the four-total, three-total, and two-total subgroups, there is a definite configuration that performs the best, this being the (2-2), (2-1), and (2-0) configurations, where (c-r) are the number of cameras and radars respectively, which show a RMSE decrease of 22.231, 36.106, and 95.827 respectively. For combinations with a low number of sensor, the percentage decrease is dramatic. This is expected, as greater care must be taken when placing few sensors to ensure that the placement allows the sensors to work with their strengths. This is most notable in the two-total subgroup.

**Figure 6:**
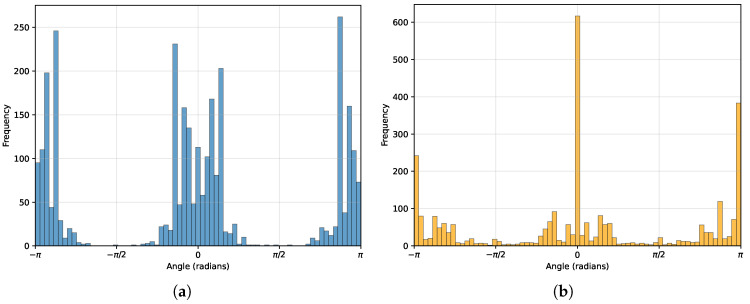
Histogram plots of the final azimuth angle (in radians) of the sensors in a (2-2) configuration, (**a**) is the camera’s final azimuth, and (**b**) is for the radar’s final azimuth. The camera’s histogram shows that there are broader groups that the cameras tend towards. This is not the case for the radars, where there is a greater, single azimuth angle that the radars move towards. This could be due to the radar measuring the balls’ azimuth, and thus need to be in a position where this measurement can be fully utilized. An interesting note is that the cameras and radars tend towards the same azimuth points, which are on either side of the length of the pitch.

An interesting note is that, in purely radar systems, a large percentage decrease occurs. This implies that the placement of the radars purely radar systems is vital for performance and decreasing the RMSE. The initial random placement for the radars produces an RMSE with a large mean and standard deviation. This could occur if the radars are placed close together, making their azimuth measurements too similar which can bias the mean estimate towards the radars. After the optimization is performed, the radars still produce the highest RMSE; further investigation is required to discern why this is the case. This can also be seen in Figure 6, where there are distinct positions that the radars tend towards, most notably pointing down/up the pitch. The cameras have a similar placement in Figure 6, but have a wider variance over the placement of the cameras.

Table 2 shows that there is no RMSE loss minimization metric that consistently outperforms the others. This is interesting as even though the determinant captures more of the covariance matrix, it does not produce results that outperform the trace or RMSE metrics. This makes sense given the fact that it is the RMSE that is being minimized, which only depends on the diagonal of the covariance matrix. Even though this is the case, working towards a smaller covariance ellipsoid, through the determinant, was expected to produce better results.

The results for the same number of radars and cameras show that after (2-2), there are diminishing returns, with a (2-2) configuration performing the same as the (3-3) and (4-4) configurations. However, with the (3-3) and (4-4) configurations, there is a lower RMSE standard deviation, which implies that the performance of these configurations is more consistent. This is intuitive, as the more sensors there are, the more constrained the covariance matrix is, where, eventually, the position of the sensors is redundant. This also explains why there is a small decrease in RMSE when there are more sensors present: the random initial starting positions for the sensors have a higher chance of already highly constraining the covariance of the points. In terms of practicality, having a (2-2) system offers the best performance for a minimal number of sensors, provided the sensors are optimally placed.

For most of the sensor configurations, the final RMSE standard deviations are low. This implies that there is a globally optimal configuration for the sensors, which the algorithm finds.

## 7. Conclusions

The algorithm is tested on a cricket ball tracking use case, which is typically used for assisted umpiring and match analysis in sports broadcasting. The results show that there are specific combinations and placements that offer superior performance if the sensors are placed correctly and that as the number of sensors increases, there are diminishing returns on the change of the RMSE, while finding placements of the sensors that minimize the RMSE for that specific sensor configuration.

In future work, the Doppler velocity of the radar can be incorporated into the optimization scheme. Furthermore, if specific regions require a stronger triangulation, such as the area before the cricket ball bounces and at the wickets, a weighting scheme can be used to force the sensors to place a higher priority on these regions.

## Figures and Tables

**Table 1:** Matrix norms and their geometric meaning. The loss is a function of one of these matrix norms applied to the covariance matrix of a point Σ.

Optimality	Loss Function	Geometric Meaning
D-optimality	lndet(Σ)	A measure of the “volume” of the covariance matrix
A-optimality	lntrace(Σ)	Natural log of the sum of the eigenvalues of the covariance matrix.
ine -	$\sqrt{\frac{1}{3}\mathrm{trace}\left(\Sigma \right)}$	*RMSE* of the covariance function

**Table 2:** Summary of log-normal mean RMSE and its standard deviation at the beginning and end of optimization for each sensor configuration and minimization criterion for the 4500 Monto Carlo runs. The start mean and standard deviation are from the random initial placements of the sensors, while the end mean and standard deviation are after the sensor placement optimization. The table also reports the absolute and percentage decrease in RMSE. Bold numbers show the best performing minimization criteria and best performing sensor configuration for the sensor configuration group.

Sensor Configuration		Start		Minimization	End			
No. Cameras	No. Radars	μ (mm)	σ (mm)	Criteria	μ (mm)	σ (mm)	Δμ (mm)	% Decrease
4	0	78.180	15.10	det	**70.966**	8.28	**−7.215**	**9.228**
				trc	71.070	**1.95**	−7.110	9.094
				RMSE	71.006	2.02	−7.174	9.177
3	1	78.249	18.58	det	68.426	10.27	−9.824	12.555
				trc	61.041	7.75	−17.209	21.992
				RMSE	**59.867**	**7.06**	**− 18.383**	**23.493**
**2**	**2**	81.257	19.94	det	**59.027**	10.75	**− 22.231**	**27.358**
				trc	62.020	**10.28**	−19.237	23.675
				RMSE	62.087	10.87	−19.170	23.592
1	3	90.309	18.22	det	69.291	0.99	−21.018	23.274
				trc	68.671	**0.75**	−21.638	23.960
				RMSE	**68.571**	0.76	**− 21.738**	**24.071**
0	4	1095.817	1992.16	det	**160.197**	99.95	**− 935.619**	**85.381**
				trc	161.310	102.00	−934.507	85.279
				RMSE	173.491	**96.32**	−922.325	84.168
3	0	90.608	28.94	det	77.381	8.64	−13.227	14.598
				trc	71.499	**0.65**	−19.109	21.090
				RMSE	**71.482**	1.21	**− 19.126**	**21.108**
**2**	**1**	96.442	35.12	det	63.003	13.74	−33.439	34.673
				trc	60.616	11.56	−35.826	37.148
				RMSE	**60.336**	**11.29**	**− 36.106**	**37.438**
1	2	111.013	35.56	det	73.077	**1.37**	−37.936	34.173
				trc	72.612	1.86	−38.401	34.591
				RMSE	**72.520**	1.94	**− 38.494**	**34.675**
0	3	2437.991	5890.00	det	223.184	192.45	−2214.808	90.846
				trc	235.126	214.61	−2202.866	90.356
				RMSE	**217.745**	**152.83**	**− 2220.246**	**91.069**
**2**	**0**	166.338	145.69	det	81.468	**1.97**	−84.870	51.022
				trc	**70.511**	2.18	**− 95.827**	**57.610**
				RMSE	72.179	7.74	−94.159	56.607
1	1	201.595	109.51	det	**95.421**	**2.71**	**− 106.173**	**52.667**
				trc	97.579	4.75	−104.016	51.597
				RMSE	98.460	10.21	−103.134	51.159
0	2	6135.995	7567.36	det	**494.726**	**206.24**	**− 5641.269**	**91.937**
				trc	1198.169	1464.78	−4937.826	80.473
				RMSE	771.072	231.43	−5364.923	87.434
**4**	**4**	64.521	7.38	det	60.953	**3.50**	−3.568	5.530
				trc	60.490	5.25	−4.031	6.248
				RMSE	**60.083**	5.54	**− 4.438**	**6.879**
**3**	**3**	68.766	10.28	det	62.694	**4.49**	−6.072	8.830
				trc	62.674	6.54	−6.092	8.859
				RMSE	**61.922**	6.92	**− 6.844**	**9.952**

## Data Availability

The ball trajectories are stored in *ball_state_spaces.npy*. The shape of the trajectories is (state space, ball number, time). The state space is of dimension 12, where the first three dimensions are for position (x, y, z) (m), then velocity, acceleration, and angular velocity.
